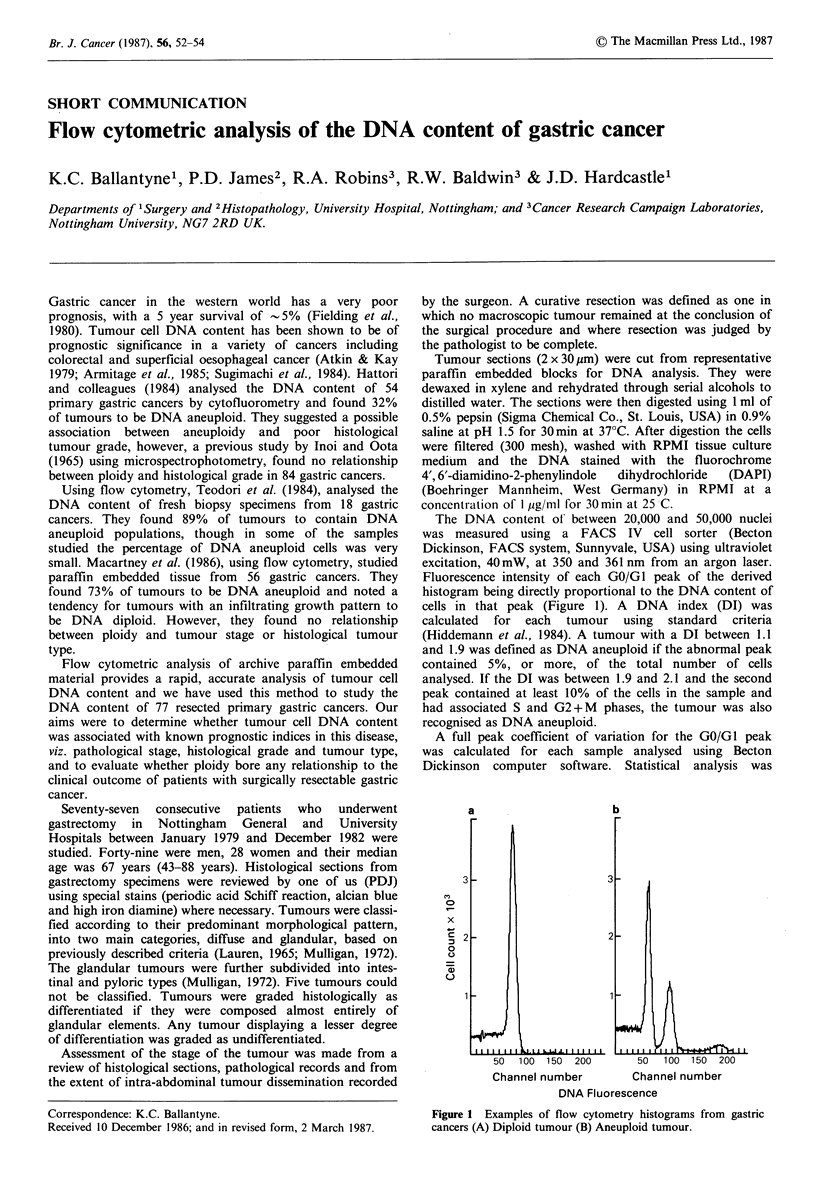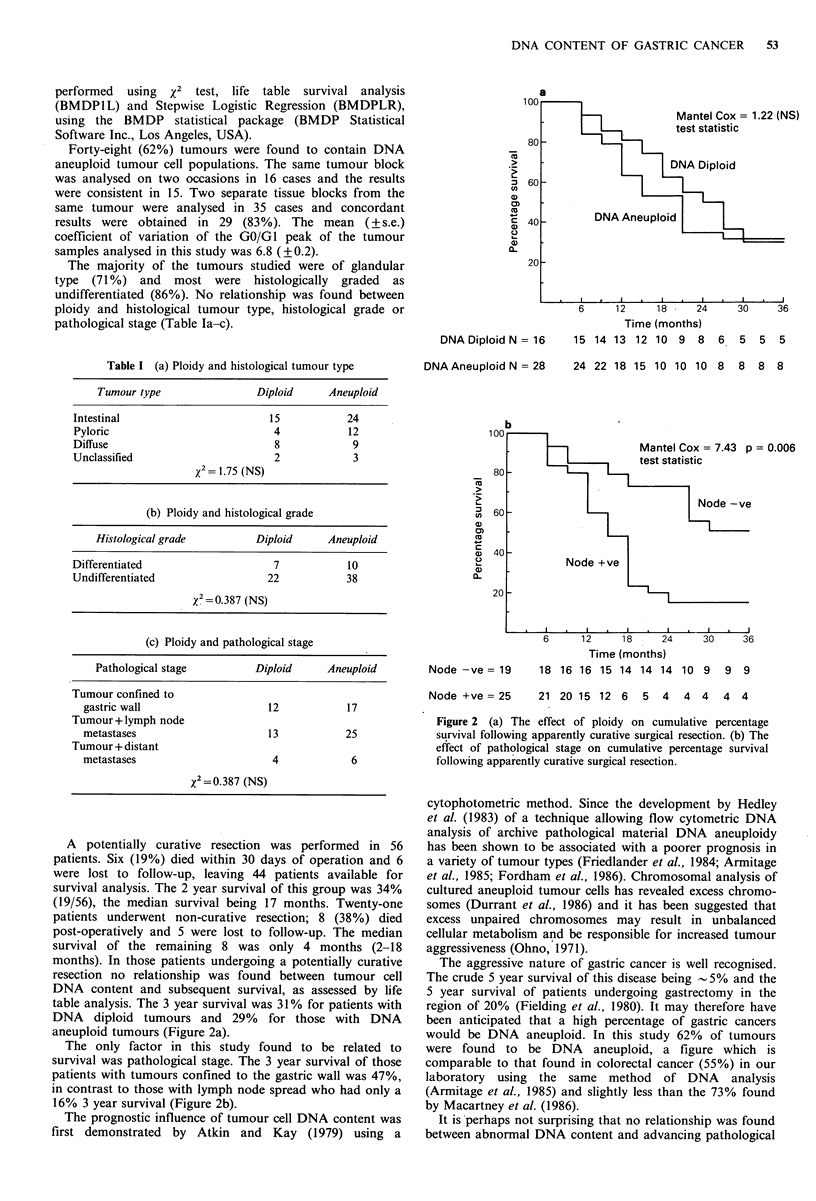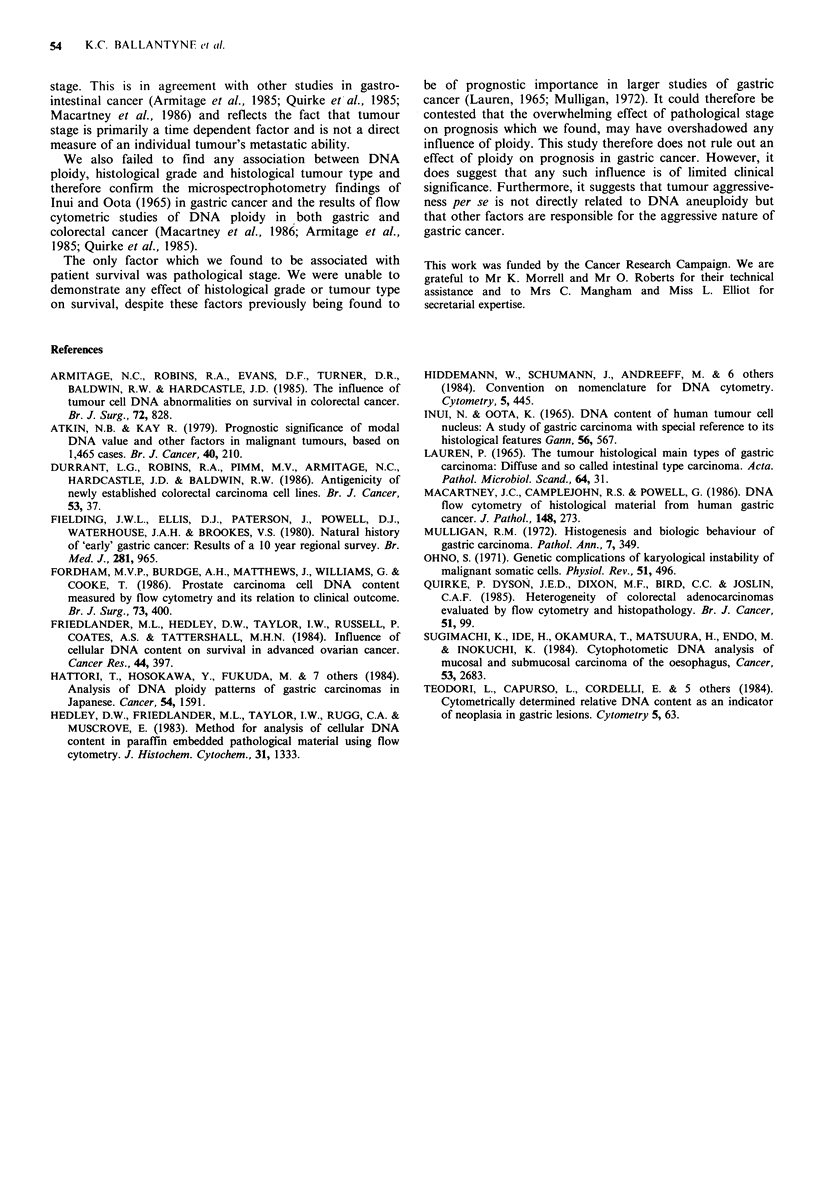# Flow cytometric analysis of the DNA content of gastric cancer.

**DOI:** 10.1038/bjc.1987.152

**Published:** 1987-07

**Authors:** K. C. Ballantyne, P. D. James, R. A. Robins, R. W. Baldwin, J. D. Hardcastle


*Br. J. Cancer* (1987), **56**, 52–54 © The Macmillan Press Ltd., 1987

SHORT COMMUNICATION

### Flow cytometric analysis of the DNA content of gastric cancer

K.C. Ballantyne[1], P.D. James[2], R.A. Robins[3], R.W. Baldwin[3] & J.D. Hardcastle[1]

*Departments of [1]Surgery and [2]Histopathology, University Hospital, Nottingham; and [3]Cancer Research Campaign Laboratories, Nottingham University, NG7 2RD UK.*

Gastric cancer in the western world has a very poor prognosis, with a 5 year survival of ~5% (Fielding *et al.*, 1980). Tumour cell DNA content has been shown to be of prognostic significance in a variety of cancers including colorectal and superficial oesophageal cancer (Atkin & Kay 1979; Armitage *et al.*, 1985; Sugimachi *et al.*, 1984). Hattori and colleagues (1984) analysed the DNA content of 54 primary gastric cancers by cytofluorometry and found 32% of tumours to be DNA aneuploid. They suggested a possible association between aneuploidy and poor histological tumour grade, however, a previous study by Inoi and Oota (1965) using microspectrophotometry, found no relationship between ploidy and histological grade in 84 gastric cancers.

Using flow cytometry, Teodori *et al.* (1984), analysed the DNA content of fresh biopsy specimens from 18 gastric cancers. They found 89% of tumours to contain DNA aneuploid populations, though in some of the samples studied the percentage of DNA aneuploid cells was very small. Macartney *et al.* (1986), using flow cytometry, studied paraffin embedded tissue from 56 gastric cancers. They found 73% of tumours to be DNA aneuploid and noted a tendency for tumours with an infiltrating growth pattern to be DNA diploid. However, they found no relationship between ploidy and tumour stage or histological tumour type.

Flow cytometric analysis of archive paraffin embedded material provides a rapid, accurate analysis of tumour cell DNA content and we have used this method to study the DNA content of 77 resected primary gastric cancers. Our aims were to determine whether tumour cell DNA content was associated with known prognostic indices in this disease, *viz.* pathological stage, histological grade and tumour type, and to evaluate whether ploidy bore any relationship to the clinical outcome of patients with surgically resectable gastric cancer.

Seventy-seven consecutive patients who underwent gastrectomy in Nottingham General and University Hospitals between January 1979 and December 1982 were studied. Forty-nine were men, 28 women and their median age was 67 years (43–88 years). Histological sections from gastrectomy specimens were reviewed by one of us (PDJ) using special stains (periodic acid Schiff reaction, alcian blue and high iron diamine) where necessary. Tumours were classified according to their predominant morphological pattern, into two main categories, diffuse and glandular, based on previously described criteria (Lauren, 1965; Mulligan, 1972). The glandular tumours were further subdivided into intestinal and pyloric types (Mulligan, 1972). Five tumours could not be classified. Tumours were graded histologically as differentiated if they were composed almost entirely of glandular elements. Any tumour displaying a lesser degree of differentiation was graded as undifferentiated.

Assessment of the stage of the tumour was made from a review of histological sections, pathological records and from the extent of intra-abdominal tumour dissemination recorded by the surgeon. A curative resection was defined as one in which no macroscopic tumour remained at the conclusion of the surgical procedure and where resection was judged by the pathologist to be complete.

Tumour sections (2 × 30 μm) were cut from representative paraffin embedded blocks for DNA analysis. They were dewaxed in xylene and rehydrated through serial alcohols to distilled water. The sections were then digested using 1 ml of 0.5% pepsin (Sigma Chemical Co., St. Louis, USA) in 0.9% saline at pH 1.5 for 30 min at 37°C. After digestion the cells were filtered (300 mesh), washed with RPMI tissue culture medium and the DNA stained with the fluorochrome 4′,6′-diamidino-2-phenylindole dihydrochloride (DAPI) (Boehringer Mannheim, West Germany) in RPMI at a concentration of 1 μg/ml for 30 min at 25°C.

The DNA content of between 20,000 and 50,000 nuclei was measured using a FACS IV cell sorter (Becton Dickinson, FACS system, Sunnyvale, USA) using ultraviolet excitation, 40 mW, at 350 and 361 nm from an argon laser. Fluorescence intensity of each G0/G1 peak of the derived histogram being directly proportional to the DNA content of cells in that peak (Figure 1). A DNA index (DI) was calculated for each tumour using standard criteria (Hiddemann *et al.*, 1984). A tumour with a DI between 1.1 and 1.9 was defined as DNA aneuploid if the abnormal peak contained 5%, or more, of the total number of cells analysed. If the DI was between 1.9 and 2.1 and the second peak contained at least 10% of the cells in the sample and had associated S and G2+M phases, the tumour was also recognised as DNA aneuploid.

A full peak coefficient of variation for the G0/G1 peak was calculated for each sample analysed using Becton Dickinson computer software. Statistical analysis was

**Figure 1** Examples of flow cytometry histograms from gastric cancers (A) Diploid tumour (B) Aneuploid tumour.

Correspondence: K.C. Ballantyne.
Received 10 December 1986; and in revised form, 2 March 1987.

performed using $\chi^2$ test, life table survival analysis (BMDP1L) and Stepwise Logistic Regression (BMDPLR), using the BMDP statistical package (BMDP Statistical Software Inc., Los Angeles, USA).

Forty-eight (62%) tumours were found to contain DNA aneuploid tumour cell populations. The same tumour block was analysed on two occasions in 16 cases and the results were consistent in 15. Two separate tissue blocks from the same tumour were analysed in 35 cases and concordant results were obtained in 29 (83%). The mean (±s.e.) coefficient of variation of the G0/G1 peak of the tumour samples analysed in this study was 6.8 (±0.2).

The majority of the tumours studied were of glandular type (71%) and most were histologically graded as undifferentiated (86%). No relationship was found between ploidy and histological tumour type, histological grade or pathological stage (Table Ia–c).

**Table I** (a) Ploidy and histological tumour type

| *Tumour type* | *Diploid* | *Aneuploid* |
|---|---|---|
| Intestinal | 15 | 24 |
| Pyloric | 4 | 12 |
| Diffuse | 8 | 9 |
| Unclassified | 2 | 3 |
| $\chi^2 = 1.75$ (NS) | | |

(b) Ploidy and histological grade

| *Histological grade* | *Diploid* | *Aneuploid* |
|---|---|---|
| Differentiated | 7 | 10 |
| Undifferentiated | 22 | 38 |
| $\chi^2 = 0.387$ (NS) | | |

(c) Ploidy and pathological stage

| Pathological stage | *Diploid* | *Aneuploid* |
|---|---|---|
| Tumour confined to gastric wall | 12 | 17 |
| Tumour + lymph node metastases | 13 | 25 |
| Tumour + distant metastases | 4 | 6 |
| $\chi^2 = 0.387$ (NS) | | |

**Figure 2** (a) The effect of ploidy on cumulative percentage survival following apparently curative surgical resection. (b) The effect of pathological stage on cumulative percentage survival following apparently curative surgical resection.

A potentially curative resection was performed in 56 patients. Six (19%) died within 30 days of operation and 6 were lost to follow-up, leaving 44 patients available for survival analysis. The 2 year survival of this group was 34% (19/56), the median survival being 17 months. Twenty-one patients underwent non-curative resection; 8 (38%) died post-operatively and 5 were lost to follow-up. The median survival of the remaining 8 was only 4 months (2–18 months). In those patients undergoing a potentially curative resection no relationship was found between tumour cell DNA content and subsequent survival, as assessed by life table analysis. The 3 year survival was 31% for patients with DNA diploid tumours and 29% for those with DNA aneuploid tumours (Figure 2a).

The only factor in this study found to be related to survival was pathological stage. The 3 year survival of those patients with tumours confined to the gastric wall was 47%, in contrast to those with lymph node spread who had only a 16% 3 year survival (Figure 2b).

The prognostic influence of tumour cell DNA content was first demonstrated by Atkin and Kay (1979) using a cytophotometric method. Since the development by Hedley *et al.* (1983) of a technique allowing flow cytometric DNA analysis of archive pathological material DNA aneuploidy has been shown to be associated with a poorer prognosis in a variety of tumour types (Friedlander *et al.*, 1984; Armitage *et al.*, 1985; Fordham *et al.*, 1986). Chromosomal analysis of cultured aneuploid tumour cells has revealed excess chromosomes (Durrant *et al.*, 1986) and it has been suggested that excess unpaired chromosomes may result in unbalanced cellular metabolism and be responsible for increased tumour aggressiveness (Ohno, 1971).

The aggressive nature of gastric cancer is well recognised. The crude 5 year survival of this disease being ~5% and the 5 year survival of patients undergoing gastrectomy in the region of 20% (Fielding *et al.*, 1980). It may therefore have been anticipated that a high percentage of gastric cancers would be DNA aneuploid. In this study 62% of tumours were found to be DNA aneuploid, a figure which is comparable to that found in colorectal cancer (55%) in our laboratory using the same method of DNA analysis (Armitage *et al.*, 1985) and slightly less than the 73% found by Macartney *et al.* (1986).

It is perhaps not surprising that no relationship was found between abnormal DNA content and advancing pathological stage. This is in agreement with other studies in gastrointestinal cancer (Armitage *et al.*, 1985; Quirke *et al.*, 1985; Macartney *et al.*, 1986) and reflects the fact that tumour stage is primarily a time dependent factor and is not a direct measure of an individual tumour's metastatic ability.

We also failed to find any association between DNA ploidy, histological grade and histological tumour type and therefore confirm the microspectrophotometry findings of Inui and Oota (1965) in gastric cancer and the results of flow cytometric studies of DNA ploidy in both gastric and colorectal cancer (Macartney *et al.*, 1986; Armitage *et al.*, 1985; Quirke *et al.*, 1985).

The only factor which we found to be associated with patient survival was pathological stage. We were unable to demonstrate any effect of histological grade or tumour type on survival, despite these factors previously being found to be of prognostic importance in larger studies of gastric cancer (Lauren, 1965; Mulligan, 1972). It could therefore be contested that the overwhelming effect of pathological stage on prognosis which we found, may have overshadowed any influence of ploidy. This study therefore does not rule out an effect of ploidy on prognosis in gastric cancer. However, it does suggest that any such influence is of limited clinical significance. Furthermore, it suggests that tumour aggressiveness *per se* is not directly related to DNA aneuploidy but that other factors are responsible for the aggressive nature of gastric cancer.

This work was funded by the Cancer Research Campaign. We are grateful to Mr K. Morrell and Mr O. Roberts for their technical assistance and to Mrs C. Mangham and Miss L. Elliot for secretarial expertise.

### References

ARMITAGE, N.C., ROBINS, R.A., EVANS, D.F., TURNER, D.R., BALDWIN, R.W. & HARDCASTLE, J.D. (1985). The influence of tumour cell DNA abnormalities on survival in colorectal cancer. *Br. J. Surg.*, **72**, 828.

ATKIN, N.B. & KAY R. (1979). Prognostic significance of modal DNA value and other factors in malignant tumours, based on 1,465 cases. *Br. J. Cancer*, **40**, 210.

DURRANT, L.G., ROBINS, R.A., PIMM, M.V., ARMITAGE, N.C., HARDCASTLE, J.D. & BALDWIN, R.W. (1986). Antigenicity of newly established colorectal carcinoma cell lines. *Br. J. Cancer*, **53**, 37.

FIELDING, J.W.L., ELLIS, D.J., PATERSON, J., POWELL, D.J., WATERHOUSE, J.A.H. & BROOKES, V.S. (1980). Natural history of 'early' gastric cancer: Results of a 10 year regional survey. *Br. Med. J.*, **281**, 965.

FORDHAM, M.V.P., BURDGE, A.H., MATTHEWS, J., WILLIAMS, G. & COOKE, T. (1986). Prostate carcinoma cell DNA content measured by flow cytometry and its relation to clinical outcome. *Br. J. Surg.*, **73**, 400.

FRIEDLANDER, M.L., HEDLEY, D.W., TAYLOR, I.W., RUSSELL, P. COATES, A.S. & TATTERSHALL, M.H.N. (1984). Influence of cellular DNA content on survival in advanced ovarian cancer. *Cancer Res.*, **44**, 397.

HATTORI, T., HOSOKAWA, Y., FUKUDA, M. & 7 others (1984). Analysis of DNA ploidy patterns of gastric carcinomas in Japanese. *Cancer*, **54**, 1591.

HEDLEY, D.W., FRIEDLANDER, M.L., TAYLOR, I.W., RUGG, C.A. & MUSCROVE, E. (1983). Method for analysis of cellular DNA content in paraffin embedded pathological material using flow cytometry. *J. Histochem. Cytochem.*, **31**, 1333.

HIDDEMANN, W., SCHUMANN, J., ANDREEFF, M. & 6 others (1984). Convention on nomenclature for DNA cytometry. *Cytometry*, **5**, 445.

INUI, N. & OOTA, K. (1965). DNA content of human tumour cell nucleus: A study of gastric carcinoma with special reference to its histological features *Gann*, **56**, 567.

LAUREN, P. (1965). The tumour histological main types of gastric carcinoma: Diffuse and so called intestinal type carcinoma. *Acta. Pathol. Microbiol. Scand.*, **64**, 31.

MACARTNEY, J.C., CAMPLEJOHN, R.S. & POWELL, G. (1986). DNA flow cytometry of histological material from human gastric cancer. *J. Pathol.*, **148**, 273.

MULLIGAN, R.M. (1972). Histogenesis and biologic behaviour of gastric carcinoma. *Pathol. Ann.*, **7**, 349.

OHNO, S. (1971). Genetic complications of karyological instability of malignant somatic cells. *Physiol. Rev.*, **51**, 496.

QUIRKE, P. DYSON, J.E.D., DIXON, M.F., BIRD, C.C. & JOSLIN, C.A.F. (1985). Heterogeneity of colorectal adenocarcinomas evaluated by flow cytometry and histopathology. *Br. J. Cancer*, **51**, 99.

SUGIMACHI, K., IDE, H., OKAMURA, T., MATSUURA, H., ENDO, M. & INOKUCHI, K. (1984). Cytophotometic DNA analysis of mucosal and submucosal carcinoma of the oesophagus, *Cancer*, **53**, 2683.

TEODORI, L., CAPURSO, L., CORDELLI, E. & 5 others (1984). Cytometrically determined relative DNA content as an indicator of neoplasia in gastric lesions. *Cytometry* **5**, 63.